# Properties of Chemically Combusted Calcium Carbide Residue and Its Influence on Cement Properties

**DOI:** 10.3390/ma8020638

**Published:** 2015-02-13

**Authors:** Hongfang Sun, Zishanshan Li, Jing Bai, Shazim Ali Memon, Biqin Dong, Yuan Fang, Weiting Xu, Feng Xing

**Affiliations:** 1 Guangdong Provincial Key Laboratory of Durability for Marine Civil Engineering, College of Civil Engineering, Shenzhen University, Shenzhen 518060, Guangdong, China; E-Mails: sunhf03@szu.edu.cn (H.S.); lzssaus@gmail.com (Z.L.); 2011040260@emails.szu.edu.cn (J.B.); incise@szu.edu.cn (B.D.); yuanfang@szu.edu.cn (Y.F.); 2 Department of Civil Engineering, COMSATS Institute of Information Technology, Abbottabad 22010, Pakistan; E-Mail: shazimalimemon@gmail.com

**Keywords:** calcium carbide residue, by-product, reactive cementitious powder, chemical combustion

## Abstract

Calcium carbide residue (CCR) is a waste by-product from acetylene gas production. The main component of CCR is Ca(OH)_2_, which can react with siliceous materials through pozzolanic reactions, resulting in a product similar to those obtained from the cement hydration process. Thus, it is possible to use CCR as a substitute for Portland cement in concrete. In this research, we synthesized CCR and silica fume through a chemical combustion technique to produce a new reactive cementitious powder (RCP). The properties of paste and mortar in fresh and hardened states (setting time, shrinkage, and compressive strength) with 5% cement replacement by RCP were evaluated. The hydration of RCP and OPC (Ordinary Portland Cement) pastes was also examined through SEM (scanning electron microscope). Test results showed that in comparison to control OPC mix, the hydration products for the RCP mix took longer to formulate. The initial and final setting times were prolonged, while the drying shrinkage was significantly reduced. The compressive strength at the age of 45 days for RCP mortar mix was found to be higher than that of OPC mortar and OPC mortar with silica fume mix by 10% and 8%, respectively. Therefore, the synthesized RCP was proved to be a sustainable active cementitious powder for the strength enhanced of building materials, which will result in the diversion of significant quantities of this by-product from landfills.

## 1. Introduction

At present, the construction industry is encountering the challenge of incorporating sustainability into their production processes, either by searching for or incorporating new raw materials and products that are more environmental friendly and/or contributing towards the reduction of CO_2_ emissions into the atmosphere. The possibility of incorporating waste from industrial or agricultural activities in their production processes can help to achieve this goal [1]. Different pozzolans, such as fly ash, silica fume, metakaolin, and rice husk ash *etc.*, are found to be viable cement alternatives [2,3,4,5]. These by-products have been found to significantly enhance the mechanical and durability properties of the resulting cementitious systems. Moreover, depending on the composition of materials, relatively denser, stronger, and stiffer composites can be obtained from these mixtures.

Calcium carbide residue (CCR) is a by-product obtained from the acetylene gas (C_2_H_2_) production process, as shown in the following equation:

CaC_2_ +2H_2_O → C_2_H_2_ + Ca(OH)_2_ [6] (1)

Acetylene (C_2_H_2_) gas is widely used for ripening fruit in agriculture and for welding in industry, while the by-product (CCR) is often discarded as waste in landfills and thus poses a threat to the environment. For example, in China, as much as 2500 tons of CCR is generated annually [7]. CCR is mainly composed of calcium hydroxide with a mass fraction of above 92% and is highly alkaline (pH > 12). It has been found that mixing CCR with certain pozzolans, which have high silicon dioxide (SiO_2_) or aluminum oxide (Al_2_O_3_) content, could yield pozzolanic reactions, resulting in final products that are similar to those obtained from the cement hydration process [8].

In order to reduce the environmental pollution, attempts have been made to utilize CCR in a better way, especially for building material applications. Since the dominant component of CCR is Ca(OH)_2_, it provides a potential application in cement manufacturing industry and can be used as a cement replacement material. In recent past, some researchers used CCR as a substitute for limestone to produce clinker [8]. CCR was also used as a cement replacement material and mixed with fly ash [6,9,10,11,12,13] or waste ashes [14,15] to produce a cementitious material. Researchers and scientists are still searching for new approaches to recycle CCR in different ways.

The purpose of this research is to recycle CCR waste by investigating the possibility of using it (in place of limestone) as a calcium oxide source for the production of clinker. The CCR is recycled by synthesizing it to a reactive cemetitious powder through a new combustion technique developed and patented in references [16,17]. This combustion approach is based on producing a clinker that contains a mixture of raw materials (which includes limestone, clay, and aluminum nitrate, *etc.*) and a fuel (urea, nitric acid, *etc.*) which can initiate combustion at a relatively low temperature (such as several hundred °C). Therefore, this technique has the potential for less energy consuming, since the mixtures could burn at a much lower temperature (usually approximately 600 to 850 °C) in the oven compared with conventional Portland cement manufacturing process (1300 to 1500 °C) [18,19,20]. Thus, the CCR recycling process contributes to encourage conservation and reduce CO_2_ emissions. After synthesizing, the morphology of raw and synthesized CCR was evaluated. The properties of paste and mortar in fresh and hardened state (setting time, shrinkage, and compressive strength) with 5% cement replacement by RCP were tested. Besides, the hydration of RCP and OPC paste was also examined through SEM.

## 2. Experimental Section

### 2.1. Materials

The following materials were used for this research: Ordinary Portland Cement (OPC), CCR, silica fume (SF), urea, nitric acid, superplastisizer (SP), and fine aggregates. CCR was obtained from Shantung Province, OPC with strength grade of 42.5 from Huarun, while SF, urea, and nitric acid were all purchased from Xilong Chemical Co., Ltd. (Shantou, China). Moreover, standard quartz sand having fineness modulus of 3.02 and specific gravity of 2.61 was used as fine aggregate while Sika ViscoCrete produced by Sika Corporation (Guangzhou, China) was used as superplasticizer. The physical and chemical properties of dry CCR, OPC, and SF are enlisted in Table 1, Table 2 and Table 3. The high contents of Ca(OH)_2_ in CCR indicates that it can react with pozzolanic material and produce a cementitious material.

**Table 1:** Chemical compositions of dry CCR.

Ingredient	Ca(OH)_2_	CaCO_3_	SiO_2_	Fe_2_O_3_	Al_2_O_3_	LOI (loss on ignition)
Content (%)	92	2.9	1.32	0.94	0.06	1.02

**Table 2:** Physical properties of CCR.

Physical Properties	Specific Gravity	Retained on Sieve No. 325 (%)	BET * Surface Area (m^2^/g)	Median Particle Size, d50 (μm)
CCR	2.92	3.50	7.05	9.05

Note: * Brunauer-Emmett-Teller.

**Table 3:** Chemical and physical properties of OPC and SF.

Chemical Composition (%)	OPC	SF
SiO_2_	22.52	94.00
Al_2_O_3_	5.80	0.21
Fe_2_O_3_	3.52	0.09
SO_3_	2.54	-
CaO	62.08	0.12
MgO	1.55	0.33
Na_2_O	0.05	-
K_2_O	0.56	0.38
LOI	0.94	1.50
Physical property	-	-
Specific gravity	3.12	2.80
Retained on sieve No. 325 (%)	4.70	1.00
BET surface area (m^2^/g)	2.70	21.08
Median particle size, d50 (μm)	12.00	3.11

### 2.2. Synthesis of RCP

**Figure 1:**
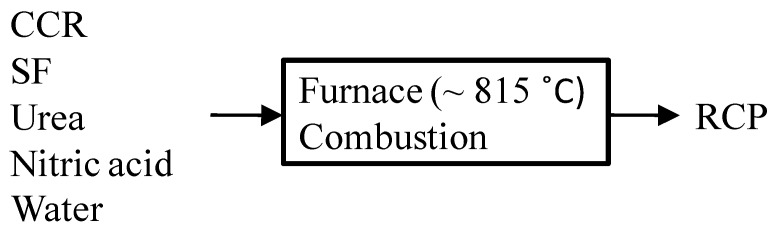
Flow chart of the RCP synthesis process.

For the synthesis of RCP, CCR and SF were used as reactants while urea with 99.5% purity and nitric acid with concentration of 65% were used as fuel to provide heat during RCP combustion. The flow chart of the one step synthesis process of RCP is shown in Figure 1. Firstly, the CCR and SF powders were mixed with the urea and nitric acid by hand with a stirrer for 1 min to form a slurry. After mixing, the slurry was fed into a furnace where the temperature was raised from room temperature to 815 °C at a heating rate of 20 °C/min. Thereafter, the system was naturally cooled down to room temperature and the final product (RCP) was collected for the subsequent tests. The detailed percentages of various materials used for synthesizing RCP are given in Table 4.

**Table 4:** Mixing proportions for synthesizing RCP.

Ingredients	CCR	SF	Urea	Nitric Acid	Water
Content (%)	10.92	4.16	47.21	23.33	14.38

### 2.3. Mix Proportion

The details of the mix proportions are enlisted in Table 5. In Table 5, OPC represents the control paste/mortar, OPC–SF represents the paste/mortar containing 10% silica fume as an additive while the last mix OPC95/RCP5–SF represents paste/mortar containing 10% silica fume as an additive and 5% RCP as replacement of cement. 

A water to cement (OPC or OPC + RCP) ratio of 0.21 was used for all mixes. Moreover, the batches were prepared by using a mechanical mixer confirming to the requirements of ASTM C305-06 [21].

**Table 5:** Mixing proportion of mortars.

Specimens	Mass (g)					
	OPC	RCP	SF	SP	Sand	Water
OPC	100	0	0	1.6	100	21
OPC–SF	100	0	10	1.6	100	21
OPC95/RCP5–SF	95	5	10	1.6	100	21

Notes: OPC: ordinary Portland cement; RCP: reactive cemenetitious powder; SF: silica fume; SP: superplasticizer.

### 2.4. Testing

The specific surface area of the raw materials as well as the synthesized RCP was measured with a Physisorption Analyzer ASAP 2020 (Micromeritics Instrument, Norcross, GA, USA) by nitrogen adsorption at 77 K using the Brunauer-Emmett-Teller (BET) method. The particle size distribution of powdered samples was measured with a Microtrac S3500 particle analyzer (Microtrac Inc., Montgomeryville, PA, USA) using ethanol as a dispersant. The mineralogical analysis was carried out by X-ray diffraction (XRD) on a D8 advance X-ray diffractometer (Bruker, Karlsruhe, Germany) with a CuKα source at 40 kV and 200 mA while the quantitative analysis was performed using the Jade program 6.5.

The morphology of powdered as well as paste samples were observed by environmental scanning electron microscopy (ESEM) on a FEI Quanta 250 instrument (FEI Inc., Hillsboro, OR, USA) equipped with a field emission gun working at 15 kV and 300 Pa.

The flow test was performed according to ASTM C230 [22], where the flow values of mixtures were recorded in the range of 105–120 mm. For the initial and final setting time of the OPC, OPC–SF, and OPC95/RCP5–SF pastes, ASTM C403 [23] was followed. The drying shrinkage of mortar samples having size of 40 × 40 × 80 mm^3^ was measured at the age of 3, 7, 14, 28, and 45 days according to ASTM C596 [24]. Three samples were used for obtaining the average value of the drying shrinkage by using the following formula (Δ*L*/*L* × 100%, where L is the original length of mortar sample while Δ*L* is the dimensional variations of length due to shrinkage at the desired ages). The compressive strength of mortars samples having size of 40 × 40 × 160 mm^3^ was tested at the age of 3, 7, 14, 28, and 45 days using a YAW-300B compression machine (Jinan shidai shijin Testing machine Group Co., Ltd., Jinan, China). For compressive strength test, the strength value represented the average of three specimens.

## 3. Results and Discussion

### 3.1. Characterization of Raw Materials

**Figure 2:**
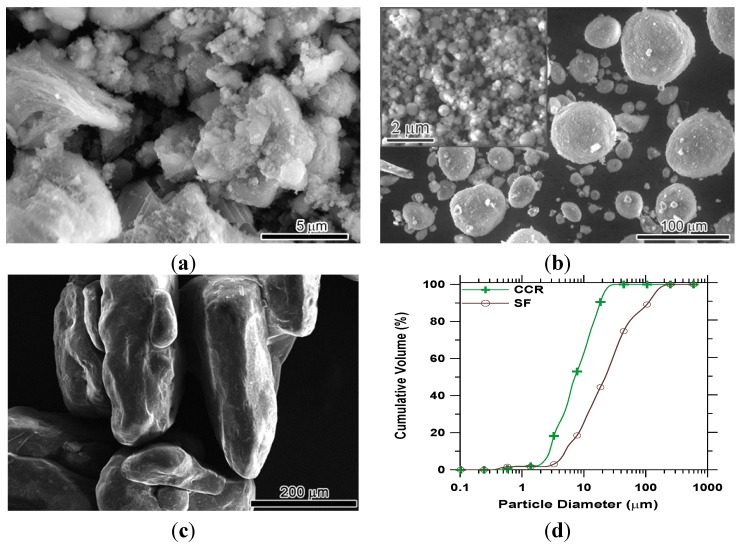
SEM images of CCR (**a**); SF (**b**); and urea (**c**); Particle size distribution of CCR and SF (**d**).

Since the properties of synthesized products are usually determined and affected by the raw materials, therefore, the raw materials used for manufacturing RCP were characterized first. The SEM images of CCR, SF, and urea particles are shown in Figure 2a–c, while the particle size distributions of CCR and SF are presented in Figure 2d. It can be seen that the CCR particles show irregular shape with a mean particle size of 9.05 μm (Figure 2a,d). SF particles appear to be round with particle sizes ranging from several to a hundred micrometers, which is consistent with particle images as shown in Figure 2b. In the enlarged view of SF particles, it can be seen that the single round SF particle appears to consist of smaller agglomerated round particles having sizes varying from tens to hundreds of nanometers (Figure 2b). The smaller size of SF particles and the pores among them contribute to the large surface area and are responsible for the high reactivity of SF. As far as urea is concerned, the microstructure shows that its size varied from sub-millimeters to millimeters (Figure 2c). The particle size distribution of urea was not measured since it dissolves in both water and ethanol dispersant. It is worth mentioning here that the BET surface area of SF and CCR was measured to be 21.08 and 7.05 m^2^/g, both of which are higher than that of OPC (2.70 m^2^/g).

### 3.2. Characterization of RCP Powder

**Figure 3:**
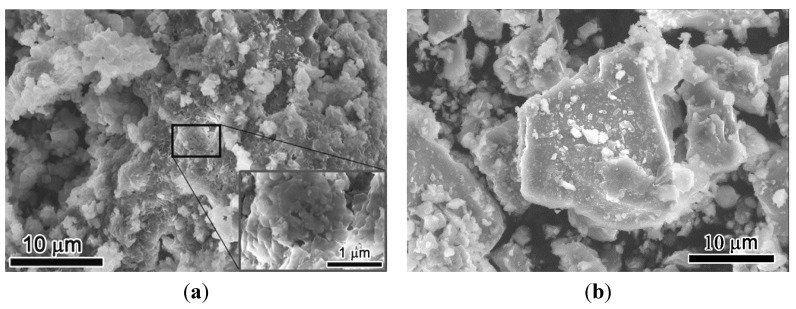
(**a**) SEM image of RCP; (**b**) SEM image of OPC.

**Figure 4:**
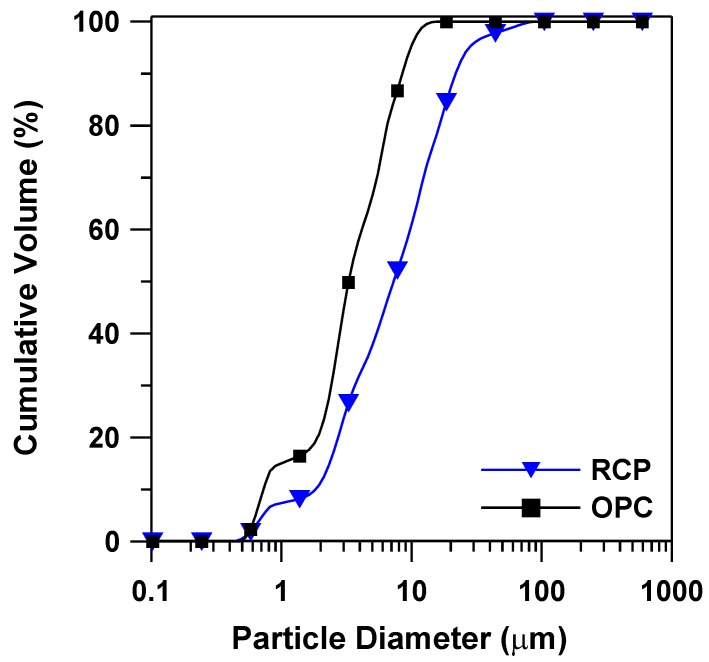
Particle size distribution of RCP and OPC powders.

The morphology and particle size distribution of RCP and OPC are shown in Figure 3 and Figure 4. The mean particle size of RCP was found to be 10.47 μm, while the mean particle size of OPC was 4.16 μm (Figure 4). Although, RCP seems to have more coarse particles than OPC, the BET surface area of the RCP was measured to be 2.04 m^2^/g, which is quite close to the measured value of OPC powder (2.70 m^2^/g). This indicates that a more porous structure exists in RCP than in OPC, which increases the surface area of RCP. It can be seen from the SEM micrographs that RCP consists of agglomerated fine particles with more porous structures (Figure 3a) than OPC powder (Figure 3b), which is in line with the testing results of the mean particle size and surface area of the powdered samples.

### 3.3. Mineralogical Analysis of RCP

**Figure 5:**
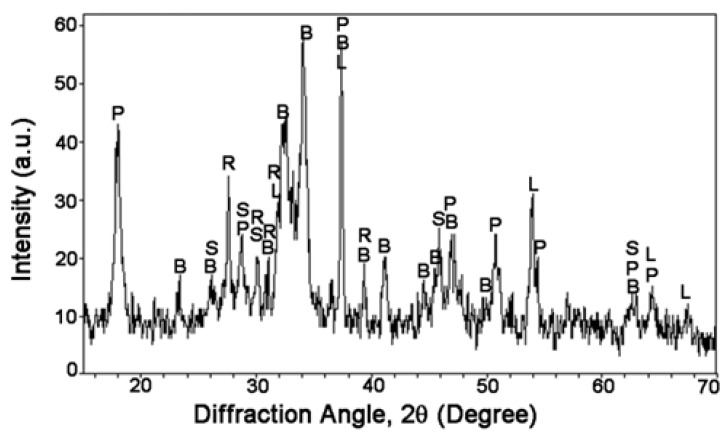
XRD pattern of RCP (B—2CaO·SiO_2_, P—Ca(OH)_2_, L—CaO, S—SiO_2_, R—Ca_3_Si_3_O_8_(OH)_2_).

The XRD pattern and the semi-quantitative compositional analysis results of RCP are shown in Figure 5 and Table 6. Belite (2CaO·SiO_2_) was found out to be the dominant component, with a mass fraction of 40.6%, followed by unreacted Ca(OH)_2_, accounting for 34.2%, and the CaO (9.3%) which may affect the hydration of cementitious matrix at the very early age. The RCP sample was also found to consist of inactive hydraulic phases (2.3% of SiO_2_ and 13.6% of hydrated Ca_3_Si_3_O_8_(OH)_2_ crystalline phase), which may have less influence on hydration.

**Table 6:** Chemical composition of RCP.

Composition	2CaO·SiO_2_	Ca(OH)_2_	CaO	SiO_2_	Ca_3_Si_3_O_8_(OH)_2_
Fraction (%)	40.6	34.2	9.3	2.3	13.6

### 3.4. Hydration Reactivity of RCP Paste

**Figure 6:**
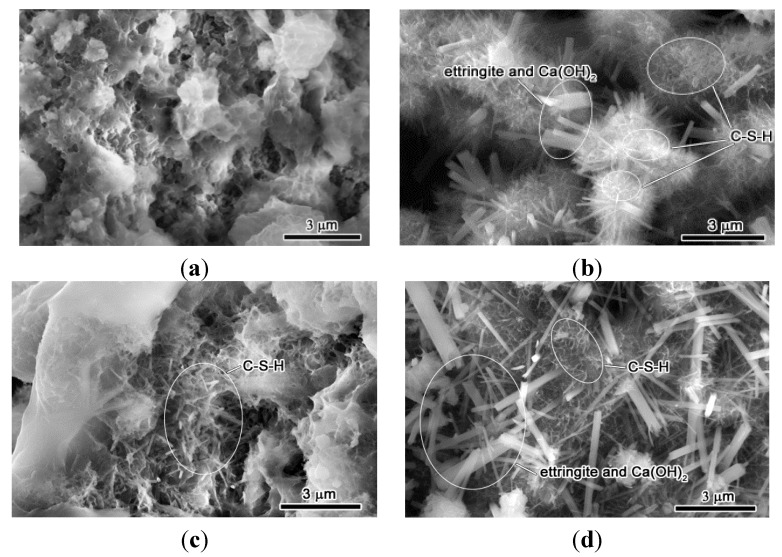

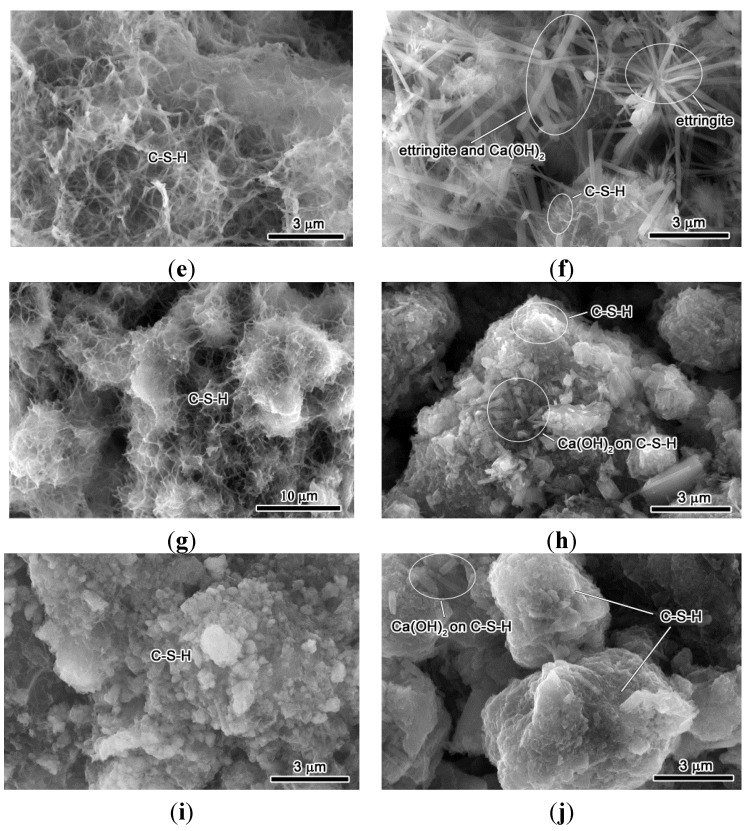
SEM observation of hydration process of RCP and OPC pastes at various ages. (**a**) RCP, 1 day; (**b**) OPC, 1 day; (**c**) RCP, 3 days; (**d**) OPC, 3 days; (**e**) RCP, 7 days; (**f**) OPC, 7 days; (**g**) RCP, 14 days; (**h**) OPC, 14 days; (**i**) RCP, 28 days; (**j**) OPC, 28 days.

Micrographs of the hydration process of the RCP and OPC paste at different ages are shown in Figure 6. All the SEM samples were taken from the fresh pastes and put directly into the ESEM chamber for *in-situ* observation. This was done so as to avoid the artificiality caused by drying and coating in normal SEM. It is seen that at the age of 1 day (Figure 6a), minor changes in morphology were observed, indicating the start of the hydration of RCP paste. For the OPC paste, the surface displays a heterogeneous distribution of fiber-like Ca(OH)_2_ and C–S–H due to the fast hydration of 3CaO·SiO_2_ and needle-like ettringite crystals from the hydration of aluminates (Figure 6b) [25,26,27]. After 3 days, fibrillar features appear in RCP paste, which are supposed to be the hydration product of 2CaO·SiO_2_ (Figure 6c). Meanwhile the length of Ca(OH)_2_ and ettringite particles of OPC paste grows and the feature of C–S–H transforms from fibrillar to foil-like (Figure 6d). The same feature transformation of C–S–H occurs to RCP paste from 3 days to 14 days with more and more of the surface was hydrated (Figure 6c,e,g). This morphology change from fibers to foils indicates the evolution from high Ca/Si (>1.5) to low Ca/Si (<1.5) in C–S–H [27]. No ettringite and Ca(OH)_2_ features were observed in RCP paste since no aluminates exist and much less Ca(OH)_2_ was produced during the hydration of 2CaO·SiO_2_ in comparison with 3CaO·SiO_2_.

Longer size of Ca(OH)_2_ and ettringite crystals was observed after 7 days in OPC paste (Figure 6f). The hydraulic OPC forms to a dense-packed cement hydration products at the age of 14 days with Ca(OH)_2_ grains dispersed on the surface of the cement matrix (Figure 6h). Moreover, a more dense and compact texture was formed at 28 days (Figure 6j). For the RCP paste, the change from the loose to dense structure occurs close to 28 days (Figure 6i). The prolonged transformation for dense structure of RCP paste compared with OPC paste is attributed to the relatively high content of 3CaO·SiO_2_ in RCP paste retarding cement hydration process at early age.

### 3.5. Initial and Final Setting Time

In order to determine the influence of RCP on the setting rate of OPC paste, the initial and final setting time of OPC, OPC–SF, and OPC95/RCP5–SF pastes were monitored. The results of these mixes are shown in Table 7. It is seen that the OPC has an initial setting time of 2.45 h and final setting time of 3.58 h. The addition of SF retarded the initial and final setting to 4.20 h and 5.28 h. When 5% of OPC was replaced by RCP, the initial and final setting time was further prolonged to 5.13 h and 6.67 h due to the slow reacting nature of 2CaO·SiO_2_ in RCP. The results are consistent with the SEM observation on hydration of pastes as reported in Figure 6.

**Table 7:** Initial and final setting time of different mixtures.

Specimen	Initial Setting Time (h)	Final Setting Time (h)
OPC	2.45	3.58
OPC–SF	4.20	5.28
OPC95/RCP5–SF	5.13	6.67

### 3.6. Drying Shrinkage

**Figure 7:**
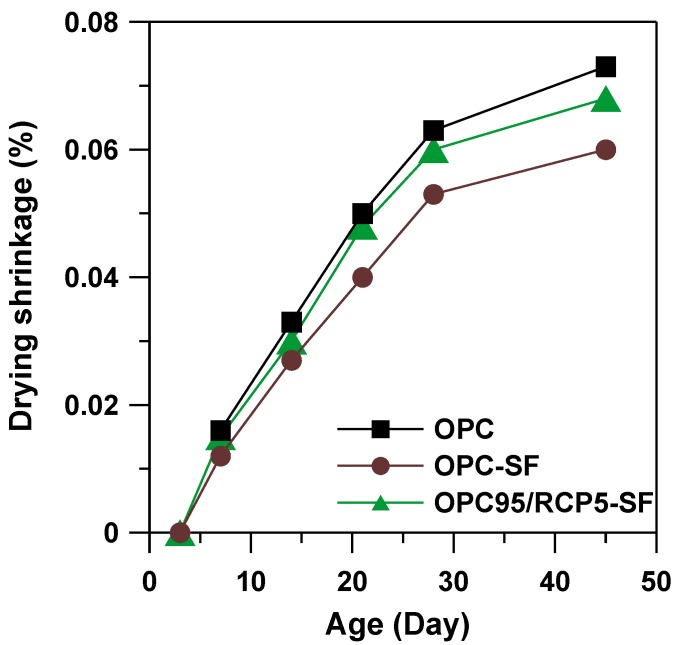
Drying shrinkage of OPC, OPC–SF, and OPC95/RCP5–SF mortars.

The drying shrinkage of RCP blended mortar was tested to reflect the influence of RCP on volume stability. The results are shown in Figure 7. The OPC mortar exhibited the largest volume shrinkage at all the testing ages. With addition of SF and RCP, the corresponding mortar exhibited less shrinkage than OPC mortar. Specifically, in comparison to OPC mortar, the shrinkage of OPC95/RCP5–SF mortar reduced by 7%. Moreover, in comparison to SF blended mortar, the OPC95/RCP5–SF mortar showed larger shrinkage values. This may be due to the volume change caused by extra hydraulic reactions between microsilica and Ca(OH)_2_ residue from the RCP manufacturing process.

### 3.7. Compressive Strength

**Figure 8:**
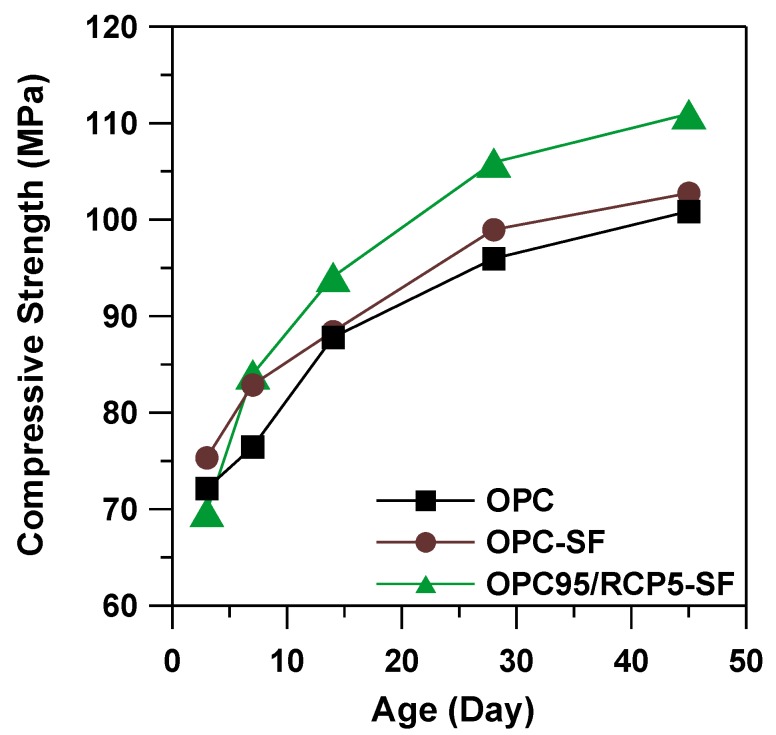
Compressive strength of OPC, OPC–SF, and OPC95/RCP5–SF mortars.

The compressive strength results of OPC, OPC–SF, and OPC95/RCP5–SF mortars mixes are shown in Figure 8. The results indicate that the compressive strength increased with increase of age for the duration of the testing. At the age of 1 day, the OPC95/RCP5–SF mortar showed the lowest compressive strength, whereas this mix showed the highest compressive strength at the age of 14 days and above. At the age of 45 days, OPC95/RCP5–SF mortar mix showed a compressive strength value of 111 MPa, which is 8% and 10% higher than that of OPC–SF and OPC mortar mixes, respectively. This shows that the addition of RCP enhanced the compressive strength of mortar at later ages.

It is interesting to note that the OPC–SF showed an insignificant increase of compressive strength compared to OPC mortar mix. It is known that the contribution of SF to the strength enhancement is due to the filler effect at the very early age and pozzolanic reaction at the later age. However, with such a low w/c, there may not be enough Ca(OH)_2_ at the later age for SF to react. Moreover, it is also known that the extent of pozzolanic reaction of SF may be determined by the amount of non-evaporable water content at any age of mortar [28]. This shows that if the amount of water is insufficient for the reaction between Ca(OH)_2_ and water to form calcium silicate hydrate, the silica reaction with Ca(OH)_2_ will not continue even at later ages of mortar [28]. Thus at a low water-binder ratio (w/b = 0.19), there may not be enough non-evaporable water in the OPC–SF mortar, which in turn, might be responsible for such a lower increase of compressive strength in comparison to OPC mortar, whereas in OPC95/RCP5–SF mortar, the RCP component has about 34.2% of residue Ca(OH)**_2_** (Table 6), which provides a second source of Ca(OH)**_2_** besides the hydration product for extra pozzolanic reaction between SF and Ca(OH)_2_. It may explain the significant increase of compressive strength of OPC95/RCP5–SF comparing with OPC and OPC–SF mortars. 

## 4. Conclusions

In this research, we investigated the feasibility of using CCR waste as a calcium oxide source for the production of clinker. The CCR was recycled by synthesizing it to a reactive cemetitious powder through a new combustion technique. The main conclusions are as follows:

1. In comparison to OPC, RCP is synthesized at quite a low temperature (approximately 800 °C). This shows the potential to reduce the energy consumption. Moreover, it would increase the sustainable value of waste material instead of sending them to landfills.
2. The BET surface area of the RCP was quite similar to that of OPC. This indicates that a more porous structure exists in RCP than in OPC, which increases the surface area of RCP. RCP consisted of 40.6% 2CaO·SiO_2_, 34.2% residual Ca(OH)_2_, and 13.6% Ca_3_Si_3_O_8_(OH)_2_. These components can potentially improve the content of hydraulic reactive constituents in cement.
3. From SEM micrographs and in comparison to OPC mix, RCP was found to retard the hydration reaction due to the slow-reacting nature of 2CaO·SiO_2_. The results are consistent with the delayed initial and final setting time results.
4. The drying shrinkage of OPC95/RCP5–SF mix reduced by 7% in comparison to the OPC mortar at the age of 45 days. Moreover, at the age of 45 days, the compressive strength of OPC95/RCP5–SF mortar mix was found to be 111 MPa, which is higher than that of OPC–SF mortar by 8% and OPC mortar by 10%, respectively. An optimization of the OPC replacement by RCP will be considered in our future research so as to enhance the compressive strength of the cementitious system.
